# Continuous Flow with Reagent Injection on an Inlaid Microfluidic Platform Applied to Nitrite Determination

**DOI:** 10.3390/mi15040519

**Published:** 2024-04-12

**Authors:** Shahrooz Motahari, Sean Morgan, Andre Hendricks, Colin Sonnichsen, Vincent Sieben

**Affiliations:** 1 Department of Electrical & Computer Engineering, Dalhousie University, 1360 Barrington Street, Halifax, NS B3H 4R2, Canada; shahrooz.motahari@dal.ca (S.M.); andre.hendricks@dal.ca (A.H.); colin.sonnichsen@dal.ca (C.S.); 2 Department of Oceanography, Dalhousie University, 1355 Oxford Street, Halifax, NS B3H 4R2, Canada; sean.morgan@dal.ca

**Keywords:** microfluidics, ocean sensors, nitrite, lab on chip, environmental monitoring

## Abstract

A continuous flow with reagent injection method on a novel inlaid microfluidic platform for nitrite determination has been successfully developed. The significance of the high-frequency monitoring of nutrient fluctuations in marine environments is crucial for understanding our impacts on the ecosystem. Many in-situ systems face limitations in high-frequency data collection and have restricted deployment times due to high reagent consumption. The proposed microfluidic device employs automatic colorimetric absorbance spectrophotometry, using the Griess assay for nitrite determination, with minimal reagent usage. The sensor incorporates 10 solenoid valves, four syringes, two LEDs, four photodiodes, and an inlaid microfluidic technique to facilitate optical measurements of fluid volumes. In this flow system, Taylor–Aris dispersion was simulated for different injection volumes at a constant flow rate, and the results have been experimentally confirmed using red food dye injection into a carrier stream. A series of tests were conducted to determine a suitable injection frequency for the reagent. Following the initial system characterization, seven different standard concentrations ranging from 0.125 to 10 µM nitrite were run through the microfluidic device to acquire a calibration curve. Three different calibrations were performed to optimize plug length, with reagent injection volumes of 4, 20, and 50 µL. A straightforward signal processing method was implemented to mitigate the Schlieren effect caused by differences in refractive indexes between the reagent and standards. The results demonstrate that a sampling frequency of at least 10 samples per hour is achievable using this system. The obtained attenuation coefficients exhibited good agreement with the literature, while the reagent consumption was significantly reduced. The limit of detection for a 20 µL injection volume was determined to be 94 nM from the sample intake, and the limit of quantification was 312 nM. Going forward, the demonstrated system will be packaged in a submersible enclosure to facilitate in-situ colorimetric measurements in marine environments.

## 1. Introduction

The accurate measurement of nitrate and nitrite at high spatial and temporal resolutions is necessary for advancing our understanding of biogeochemical nitrogen cycling within aquatic ecosystems [1,2,3,4]. Nitrate, a negative ion found in water environments, originates from various sources such as fertilizer or manure runoff, atmospheric deposition, agricultural activities, septic tanks, and wastewater treatment plants [2,3]. Nitrate, as a stable form of nitrogen in water, can be converted into nitrite nitrogen and ammonia nitrogen, leading to eutrophication and adversely affecting both human health and aquatic organisms’ growth [2,5,6]. Consequently, there is a desire for precise measurements of nitrate plus nitrite in water. Nitrate concentration in the ocean exhibits variability, ranging from 0.1–2.5 µM at the surface ocean to 2–40 µM in the deep ocean, while nitrite ranges from 0.1–200 nM at the surface ocean and 0.1–5 nM in the deep ocean [7].

Traditionally, the predominant method for acquiring ocean chemistry data involves research ships collecting individual seawater samples, which are then either analyzed on board in containerized laboratories or preserved and transported for subsequent analysis on land [8]. This methodology is not only costly and time-intensive, but also yields low-resolution data sets, leading to a substantial under-sampling of global ocean chemistry. To address this issue, various in-situ sensors have been created to enhance the spatial and temporal resolution of nutrient measurements [9,10,11,12,13]. The integration of chemical sensors into oceanographic platforms, such as floats [14], autonomous underwater vehicles (AUVs) [15], and oceanic gliders [16], facilitates the automated collection of chemical data across a range of spatial and temporal scales [17]. Sensors that are amenable to such robotic platforms must fit within restricted payload constraints, be small, exhibit low reagent consumption, minimize power usage and have capabilities to ensure data quality such as on-board calibration standards.

Various well-established techniques are available for measuring nitrate and nitrite, encompassing spectroscopic [18], electrochemical [19,20,21], and biosensor [22,23] methods. Spectroscopic approaches comprise reagent-based wet chemistry, such as colorimetry based on the Griess assay [24], chemiluminescence [25], fluorescence [26], and ultraviolet (UV) absorption [27]. UV absorption in-situ nitrite sensors have gained widespread use in both academic and industrial settings due to their lack of moving parts, minimal consumables, and high-frequency data. ISUS (In-situ Ultraviolet Spectrophotometer) developed by the Monterey Bay Aquarium Research Institute (MBARI) [28], Submersible Ultraviolet Nitrate Analyzer (SUNA) manufactured by Sea-Bird Scientific [29], and the OPUS sensor made by TriOS GmbH [30] are examples of commercialized nitrate sensors that have been implemented on profiling floats. In the SOCCOM project [31], a large array of profiling floats has been deployed, featuring both the SUNA and ISUS nitrate sensors to monitor the dynamics of the Southern Ocean. Between August 2008 and June 2013, six profiling floats were equipped with nitrate UV and oxygen sensors and strategically deployed to monitor the Gulf of Alaska [32]. Although significant progress has been made in obtaining several high-quality datasets [15,27,33,34], it is worth noting that direct UV absorption systems face challenges such as lower performance compared to most wet chemical systems [17]. For example, the limit of detection (LOD) for SUNA v2 is 0.4 µM [35], compared to 20 nM for a wet chemical system [17]. Susceptibility to interferences [36], potential drift issues [37], and window aging/biofouling [11] are other drawbacks of UV absorption sensors.

Automated submersible flow-injection systems date back to as early as 1986, when Johnson et al. [38] introduced the SCANNER to perform the spectrophotometric detection of silicate and sulfide. Since then, the research community has reported other instances of commercially produced in-situ wet chemical systems that have been successfully deployed and documented [13,39,40]. While wet chemistry analyzers have been recognized for their stability, reliability, calibration with standard samples, and low LOD, their deployment has been hindered by certain challenges. These include their considerable physical size, higher power consumption than purely optical systems, and operator complexity, whilst using substantial reagent volumes [41]. The application of microfluidic systems aims to address several of these challenges, offering advantages such as reducing reagent and power consumption, and smaller sizes allowing for extended deployment on various oceanographic platforms like floats and gliders [8,42,43,44,45]. Eventually, the overall cost of microfluidic systems may become more affordable than their “macro-scale” counterparts through mass production of microfluidic chips [41].

An early application of microfluidics to nitrite analysis dates back to 1999 when Greenway et al. used a micro flow injection system (μ-FIA) with a spectrophotometric detection method and electro-osmotic flow [46]. Since then, many research groups worldwide have advanced the development of these systems, finding broad applications, particularly in ocean nutrient detection [47,48,49,50,51,52]. In 2010, Sieben et al. explored the use of an integrated tinted absorbance cell in lab-on-chip (LOC) systems for in-situ absorption measurements [1]. Their work utilized the Griess reaction to detect nitrite within the range of 50 nM to 10 μM, achieving a limit of detection of 14 nM [1]. In 2015, Yücel et al. applied the same tinted absorbance cell technology to obtain in-situ calibrated time series data for nitrate + nitrite at a depth of 170 m on the sea floor [53]. In 2017, Beaton et al. reported a lab-on-chip colorimetric sensor for the in-situ measurement of nitrate in meltwater rivers draining the Greenland Ice Sheet [54]. Their system demonstrated a limit of detection of 25 nM and a linear dynamic range up to 350 μM. In 2018, Vincent et al. reported the deployment of an LOC nitrate sensor integrated into an autonomous underwater vehicle (AUV) for the continuous in-situ measurement of nitrate + nitrite throughout the spring bloom duration [55]. In 2022, Beaton et al. reported phosphate and nitrate measurements obtained at deep ocean depths (>4800 m) using an LOC colorimetric analyzer [8]. The systems demonstrated by the team at NOC are available for sale by Clearwater Inc., UK. In 2023, Luy et al. combined nitrate and phosphate on a single chip, demonstrating multi-species detection on a single microfluidic device [3]. The inlaid nutrient multi-species systems are sold by Dartmouth Ocean Technologies Inc., Canada. Although microfluidic LOC systems have enabled longer deployment at lower cost, there is still a need for a significant improvement in the sampling speed of these systems to enable higher throughput at high spatial resolutions [3,8]. 

Accordingly, in 2019, Nightingale et al. reported an ultrafast in-situ nitrate and nitrite sensor based on droplet microfluidics [42]. In their system, the sample and reagents were transported through discrete droplets within an oil flow using peristaltic pumps. Remarkably, the system achieved a high measurement frequency of 8000 samples per day (~330 per hour) at River Itchen, UK, while consuming only 2.8 mL of fluid daily. These systems are commercially available and sold by Southwest Sensors (SWS), UK. However, given the small droplet sizes, the absorbance path is small, on the order of 0.5 mm, permitting a modest limit of detection of 1700 nM—suitable for riverine or high concentrations, but less suitable for the low concentrations often found in marine settings. While droplet systems show great promise for their measurement frequency, ongoing research into cavity ring down approaches [56] or other path length enhancements is needed to improve the LODs for the lower range of ocean concentrations [57].

Here, we describe a fully automated and continuous-flow nitrite analyzer based on a novel inlaid microfluidic absorbance cell that permits nanomolar detection limits with minimal reagent [58,59]. We have improved the inlaid method from our prior reports by moving from large geometric shapes that completely obstruct the optical channel [58] to the small annular approach described here. The new inlaid design is simpler to manufacture and allows for improved fabrication quality control, as more of the microfluidic channel structure is clear and visible, allowing one to identify proper bonding (no channel collapse or delamination). In the previous design [58], the entire optical channel was embedded within the black poly(methyl) methacrylate (PMMA) inlay. This made visual inspection impossible with the naked eye or microscope in order to confirm a high-quality bond (i.e., no delamination) during fabrication. However, this new design minimized the black PMMA inlay for creating an absorbance cell, and it leaves the majority of the optical channel in clear PMMA. This facilitates visual inspection to ensure a completely bonded channel structure. Like our original inlaid approach [58], the new optical cell reported here allows for the independent placement of optical components without the need for epoxy or embedding during the fabrication process of the chip. We characterize this new inlaid optical method by interfacing the microfluidic chip with a sensor instrument equipped with four syringe pumps, ten valves, and four optical measurement channels. This design offers flexibility for exploring various flow strategies, including continuous flow, flow injection, or segmented flow analyses. For demonstration, the widely recognized Griess assay was employed in a pressure-driven FIA approach to measure the concentration of nitrite standards (0.125–10 µM) and generate several optimization calibration curves. Compared to our previous design [58], employing the FIA approach reduced the reagent consumption from 0.5 mL to 4–50 µL. Therefore, the system’s compact size, automation, high-frequency data acquisition, and lower reagent consumption make it an ideal choice for future in-situ nutrient measurements.

### Theory

In the above reagent-based microfluidic colorimetric systems, for both flowing and stop–flow architectures, the sample is mixed with developing reagents and results in color development. The optical absorption observed is proportional to the analyte concentration, as described by the Beer–Lambert law. Stop–flow microfluidic systems [3,54,59] take several minutes per measurement, as the sampling rate is limited by the time needed for color development in a static optical cell [46,60,61,62]. Continuous-flow microfluidic systems [42,46] allow for more rapid and frequent measurements, on the order of seconds, since the reagent color development occurs in a long delay serpentine loaded with sequential samples [39,42,63]. Flow injection analysis (FIA) represents a common variant of continuous flow, wherein a fixed volume of reagent is injected into a carrier stream of sample [41,64,65,66,67]. This approach offers a higher throughput of 30 to 60 samples per hour [41], and lower reagent consumption on the order of a few microliters per measurement in a flowing stream, compared to stop–flow systems that often flush the entire system, requiring hundreds of microliters.

To perform flow injection analysis, small plugs of reagent are injected into a carrier stream of sample. Color will begin to develop as the plug flows through a long delay serpentine and will eventually be detected at the absorbance cell. The axial velocity profile of the pressure driven flow will be parabolic, with the highest velocity at the center of the channel. As molecules travel through the system, the non-uniform velocity profile leads to dispersion, known as Taylor–Aris dispersion. The Taylor–Aris dispersion limits the performance of continuous flow systems through sample smearing. The modified dispersion coefficient for square or rectangular channels can be modeled as:

(1) $$K=D_{m}\left[1+\frac{1}{210}f\left(\frac{d}{W}\right){\left(\frac{Ud}{D_{m}}\right)}^{2}\right]$$

In the above expression, $D_{m}$ is the molecular diffusion constant, d and W are the depth and width of the channel, and U is the mean flow velocity. The function $f\left(\frac{d}{W}\right)$ is a geometry-dependent function used to determine the effects of the side walls of the channel on the overall dispersion. The dispersion coefficient will determine the rate at which the injection plug of reagent will disperse into the surrounding carrier stream. The system requires intentional design so that the reagent plugs can be injected with as high a frequency as possible without overlapping (or at least separable by deconvolution) before they reach the absorbance cell. 

The dispersion coefficient, K, will affect the concentration impulse function, which determines the shape of the plug as it flows through the channel:

(2) $$C\left(z,t\right)=\frac{1}{\sqrt{4\pi Kt}}e^{\frac{-z^{2}}{4Kt}}$$

From the reference frame of the center of the plug, the dispersion will be symmetric about the center point. However, from the reference frame of the detector, there will be a “tail” because the plug will continue to disperse as it flows through the cell. To model the shape of output signal as measured by the detector, the input plug profile (a single square peak with width of L and height of $C_{0}$) must be convolved with concentration impulse function. If $z=x_{D}-Ut$, the output signal would be calculated as [68]:

(3) $$output\left(z,t\right)=Input\left(z\right)*C\left(z,t\right)$$

(4) $$output\left(x_{D},\;t\right)=\frac{C_{0}}{2}\left[erf\left(\frac{x_{D}-Ut+\frac{L}{2}}{\sqrt{4Kt}}\right)-erf\left(\frac{x_{D}-Ut-\frac{L}{2}}{\sqrt{4Kt}}\right)\right]$$

where $x_{D}$ is the distance from the detector. In this way, the modeled dispersion can be directly compared to the signal measured at the output of the absorbance cell. 

## 2. Materials and Methods

### 2.1. Chemical and Reagent Preparation

All chemicals and reagents were supplied by Fisher Chemical (Springfield Township, NJ, USA), unless otherwise stated. The continuous flow system was first characterized using diluted red food dye (commercial food coloring, Club House Canada) that had a peak absorbance near that of reacted nitrite. The dye was diluted with Milli-Q water to a concentration of 0.05% *v*/*v* before use. Nitrite standards were prepared via stepwise dilution of a 1000 μM nitrite stock, made from 0.069 g of sodium nitrite (NaNO_2_, CAS-No: 7632-00-0, EMD Millipore, Darmstadt, Germany) added to Milli-Q to a total volume of 1 L. The stock was then diluted to create nitrite concentrations ranging between 0.125 μM and 10 μM. Standards were stored in a dark cabinet at room temperature when not in use. The Griess reagent was prepared by combining 0.5 g of sulfanilamide, 5 mL of concentrated HCl, and 0.05 g of NEDD (N(1-Naphthyl)ethylenediamine dihydrochloride), mixed with Milli-Q to create a final volume of 500 mL. The reagent was then allowed to cool to room temperature after the addition of HCl. All chemicals were of analytical grade, and the reagents were stored near 4 °C in a dark refrigerator between uses.

### 2.2. Microfluidic Chip with Novel Inlaid Optical Cell Fabrication

The substrate of the microfluidic chip was manufactured from two discs of clear PMMA (0A000, Acrylite, Sanford, ME, USA) bonded together using a combination of chloroform vapor exposure, pressure, and heat. Cylinders of opaque black PMMA (9M001, Acrylite, Sanford, ME, USA) were inlaid into each disc prior to bonding to create an aperture to reduce the effect of background light and scattering within the microfluidic chip. The opaque inserts were cut from a 3 mm-thick sheet of acrylic using a 50 W laser cutter (Mini Helix, Epilog Laser, Mississauga, ON, Canada). Each insert was 11 mm in diameter, with a 5 mm diameter central hole in the inserts designated for the top plane. Before bonding, the inserts were sanded with a 320 grit sandpaper for 2–3 min in a circular fashion, followed by scrubbing with a Scotch pad using water and detergent until no burrs were observable on the edges. The inserts were then rinsed with Milli-Q water and dried with compressed air and IPA (isopropyl alcohol) [58]. After cleaning and sanding, the cylindrical inserts were exposed to chloroform vapor for 45 s before being press-fit into cavities that had been milled into a 9-inch by 12-inch clear acrylic sheet. The sheet was then pressed and heated in a PCB press (Multipress II, LPKF Laser & Electronics, Garbsen, Germany) using 30 kN of force and a target temperature of 116 °C for 3 h. The sheet was then left in the press for a further 3 h, with pressure maintained but heating turned off to allow the sheet to cool to room temperature. The sheet was then removed from the press and prepared for milling and engraving. 

Before milling in the surface features and through holes, the entire sheet was back-planed down by 0.3 mm using a micromill (S103, LPKF Laser & Electronics, Garbsen, Germany) to ensure that the two faces were parallel. Next, the sheet was transferred to a similar micromill (S104, LPKF Laser & Electronics, Garbsen, Germany) and the channels and absorbance cells were created using a 500 µm flat-end mill, with a prescribed depth of 500 µm. A 45-degree endmill was used to engrave the microprisms at either end of the absorbance cells, within the aperture created by the opaque inlays, cut to 0.8 mm deep. After each face of the acrylic sheet had been milled and all the features created, the sheet was transferred back to the laser cutter and the chip halves were cut out from the larger sheet as discs. Each disc underwent a pre-treatment process of sanding and cleaning similar to that used for the black PMMA inserts, followed by chloroform exposure for 45 s. They were then manually slotted into a custom alignment device and inserted into a PCB press (MultiPress S, LPKF Laser and Electronics, Garbsen, Germany) to be pressed for 2 h at 85 °C using a pressure of 625 N/cm^2^. 

### 2.3. System Design and Testing Apparatus

#### 2.3.1. Chip Design

**Figure 1:**
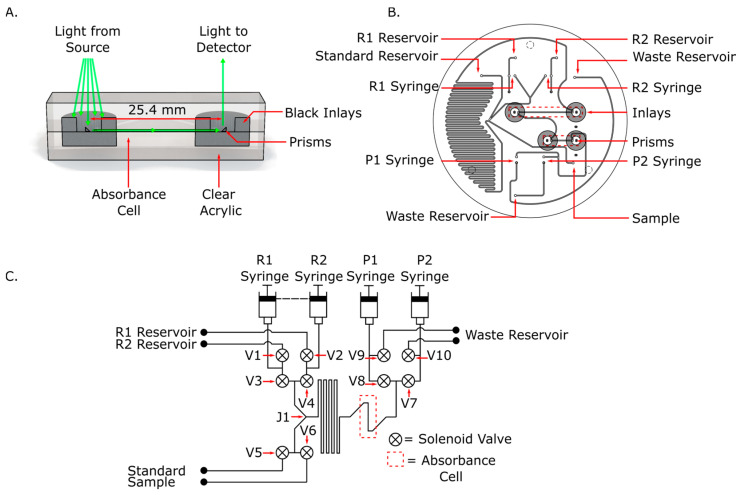
(**A**) Concept model for the improved inlaid absorbance cell, with light coming from an external source and directed through the cell to a detector. (**B**) CAD of the microfluidic chip for continuous flow analysis. The interfacing ports to the syringes are labeled R1, R2, P1 and P2, corresponding to the syringes in panel (**C**). The engraved microprisms are at the center of the inlaid circular apertures. (**C**) Fluid diagram for continuous flow on a microfluidic system. Syringes R1 and R2 contain the reagent that is periodically injected into the fluid stream in pre-programmed volumes. Syringes P1 and P2 alternate between pulling fluid through the system from either the standard or sample port and pushing fluid out through the waste ports.

The microfluidic chip was designed to perform continuous flow analysis using a push-pull configuration, built around the new inlaid absorbance cell. Figure 1A is a concept image of the inlaid absorbance cell. The two opaque cylindrical inserts act as apertures and restrict the amount of light that is able to enter or exit the absorbance cell. The engraved prisms direct light from the source above the chip, through the cell within the chip, and then back up to the detector, also outside the chip. Any external ambient light or non-collimated light form the LED is absorbed in the black inserts, limiting the light at the detector to only the light that has passed through the absorbance cell. 

Figure 1B is a CAD image of the microfluidic chip, displaying the absorbance cell and the fluid ports. The ports that interface with the syringes are labeled R1 and R2 for the two reagent syringes, and P1 and P2 for the two waste syringes. The ports that lead to standards and reagent fluids are also labeled on the diagram. The inlaid apertures and engraved microprisms are also shown in the center of the chip. The serpentine mixing chamber is 1.01 m long, resulting in a total volume of 250 µL. This volume was specifically chosen so that one stroke of a 500 µL syringe would be more than enough to carry the reagent plug all the way through the serpentine mixer and absorbance cells. 

#### 2.3.2. System Design

Figure 1C is a fluid schematic for the microfluidic device and the required syringe pumps and solenoid valves. The chip was designed to function in continuous mode, meaning that one syringe (P1 or P2) pulls the carrier stream through the chip while the reagent syringes (R1 and R2) inject small plugs of reagent into the carrier stream at junction 1 (J1). The injected plug reacts with the sample as it travels through the serpentine mixer and into the absorbance cells. In this setup, the reagent syringes were filled with the Griess reagent and injected reagent with the same flow rate as the carrier stream. However, these two syringes can be used to enable two reagent assays such as the phosphomolybdenum blue (PMB) assay for phosphate determination. Syringes P1 and P2 were operated in a push–pull configuration, wherein one syringe pulls the carrier stream through the chip while the other ejects its contents to waste, and then they switch roles at the end of their respective strokes. For controlling the fluid path, active valves (symbolized as circles with an “x”) must be opened and closed at prescribed times. For instance, in Figure 1C, V5 and V6 were used to select either the environmental sample or calibration standards as carrier streams. Table 1 describes the complete continuous flow protocol with reagent injection based on the valves and syringes shown in Figure 1C.

**Figure 2:**
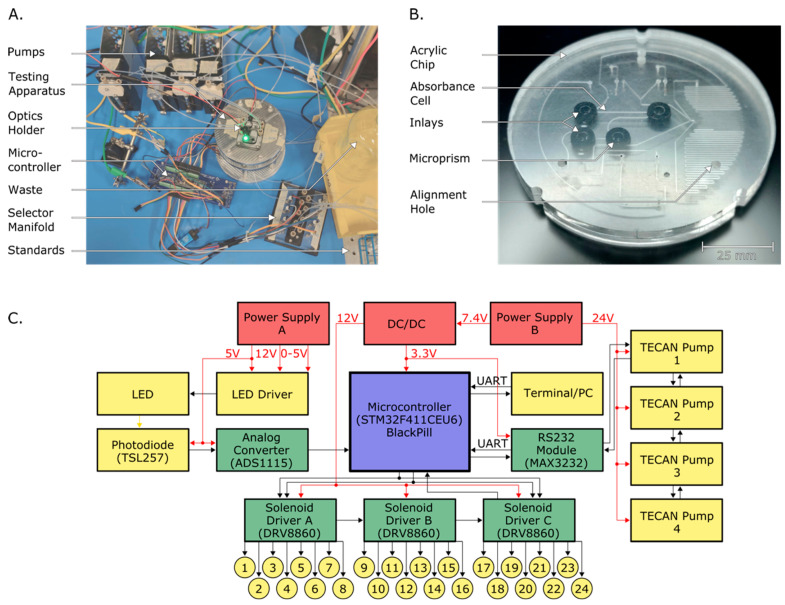
(**A**) Photograph of the bench-top testing setup for the continuous flow system. Four individually programmed Tecan syringe pumps were used to move fluid through the system, and ten solenoid valves were mounted to the testing apparatus to enable fluid control. A separate custom-made selector manifold was used to switch between fluids for automated calibrations. A 3D-printed alignment piece was used to hold the LEDs and photodiodes in position above the source and detector prisms, respectively. All electronics were controlled by a microcontroller programmed in C. (**B**) Photograph of the microfluidic chip with circular inlays. Each absorbance cell contained two inlays, one at the detector side and one at the source side. Three asymmetrical alignment holes were used to ensure correct orientation and positioning within the testing apparatus or in situ instrument. (**C**) Electrical block diagram for the testing apparatus. An STM32 BlackPill microcontroller and a custom PCB were used to automate and coordinate the electronic components of the system. Three solenoid drivers were used to control up to 24 solenoid valves, with only 10 valves used for this work. An external PC was used to create scripts and program the microcontroller to test configurations.

A custom testing apparatus was designed and fabricated to enable easy benchtop configurability and access. Figure 2A is a photograph of the benchtop setup. A stainless-steel frame was made to clamp the microfluidic chip to an acrylic valve manifold. The steel frame also contained access ports for the syringe and reagent lines, as well as vias for optical components. All fluid lines were made from polytetrafluoroethylene (PTFE) tubing with an inner diameter of 0.030 inches, and polyetheretherketone (PEEK) fittings (P-359X, IDEX Health and Science, Oak Harbor, WA, USA). A 3D-printed optical housing was bolted to the top of the frame to hold the LEDs and photodiodes in place. The frame could be disassembled via six bolts on its perimeter, which allowed the microfluidic chip to be easily replaced for rapid testing and optimization. Figure 2B is a photograph of the chip contained within the testing apparatus. The alignment holes milled in the top and bottom surfaces of the chip were used to correctly orient the chip within the frame.

Four syringe pumps (P/N 733085-B, Tecan Systems, San Jose, CA, USA) with 500 µL syringes, along with 10 solenoid valves (P/N LFNA1250125H, Lee Company, Westbrook, CT, USA), were used for fluid handling in the system. The syringe pumps interfaced with the microfluidic chip via tubing connected to the chip through ¼-28 threaded ports in the testing apparatus. The valves were mounted on a custom acrylic manifold that was clamped to the chip and sealed using o-rings. A separate selector valve manifold containing an additional eight solenoid valves was created to enable automation when switching between standards. 

All the valves and pumps were controlled using an STM32 BlackPill microcontroller programmed in C and a custom printed circuit board (PCB) described elsewhere [69]. Figure 2C is an electrical block diagram of the system. The PCB contained the microcontroller, three solenoid drivers for valves, and an analog to digital converter to read the incoming signal from the photodiodes. Communication to the syringe pumps was established using RS232 and the required UART protocol. Another UART connection was used to interface between an external computer and the microcontroller. The LED intensities were controlled using an external constant current LED driver.

### 2.4. Simulation and Dye Testing Methods

Before moving forward with reagents and analytes, dye tests and simulations were first carried out to examine the performance of the system without the added complexity of reaction kinetics. Using the models for Taylor–Aris dispersion in a microchannel, simulations using 10 µL, 50 µL and 100 µL injection volumes with a flow rate of 100 µL min^−1^ were carried out in MATLAB (R2021b). The diffusion coefficient of the dye molecules in water was unknown and was not readily found in the literature. In our Taylor–Aris calculations, we adjusted the diffusion coefficient to minimize error between the simulated peaks and the observed peaks. Interestingly, the diffusion coefficient of the dye was determined to be 1.7 × 10^−9^ m^2^ s^−1^, close to that of nitrite molecules in water [70]. According to Equation (3), the dispersion coefficient of nitrite will be 5.47 × 10^−5^ m^2^ s^−1^. The simulations were performed from the reference frame of the detector rather than the injected plug so they could be effectively compared to the signal received from the dye tests. A 0.05% *v*/*v* solution of red food dye mixed with Milli-Q was prepared and injected into the system with volumes matching the simulations. A combined flow rate of 100 µL min^−1^ was chosen for the pumps because it was the slowest allowable rate with the syringes available. The simulated signals were normalized to match the magnitude of the physical signals so as to directly compare them.

After the initial characterization with simulations and dye, a series of tests were performed to determine a suitable injection frequency for the reagent. For this test, 50 µL was chosen as the maximum total reagent (dye) injection volume (25 µL from each syringe), but characterizations at lower volumes were also carried out. Again, a flow rate of 100 µL min^−1^ was used for all the tests. To determine a suitable injection frequency for the 50 µL injection volume, a 0.05% *v*/*v* concentration of red food dye was injected at the following specified intervals: 30 s, 60 s, 80 s, and 100 s. The output photodiode signal was monitored to determine if there was smearing observed between injections. The diffusion coefficient for nitrite has been reported to be 1.7 × 10^−9^ m^2^ s^−1^ [70]; therefore, its dispersion coefficient will be 5.47 × 10^−5^ m^2^ s^−1^ using the Equation (3). 

### 2.5. Nitrite Calibrations Methods

The evaluation of the FIA inlaid design involved the injection of functional reagents into a carrier stream composed of standards with prescribed nitrite concentrations. A simple script was used to run the calibration tests. Seven different standard concentrations ranging from 0.125 to 10 µM nitrite were used as the carrier stream flowing at 100 µL min^−1^. For each standard, a plug of reagent with a pre-set volume was injected four times to ensure repeatability. Based on the results of the interval testing, only one reagent injection per syringe stroke was feasible, so between each reagent injection the system was flushed with 500 µL of the carrier stream fluid. The voltage signal was monitored as the reagent plugs reacted with the carrier stream and flowed through the absorbance cell. The measured voltage was filtered by a moving average with 5 points (at 1 Hz) and was then used to determine the absorbance. The absorbance peaks were then integrated using a custom MATLAB (R2021b) script to increase the sensitivity by monitoring the full color development as the plug flowed through the cell. The integral of the absorbance signal was calculated using the trapezoidal rule, considering a data frequency of 1 Hz. The expression for the integral is defined as:

(5) $$I_{ij}=\frac{1}{2}\sum_{n=1}^{300}A_{ij}\left(n\right)+A_{ij}\left(n+1\right)\;\;\;\;\;\;1\leq i\leq 4,\;1\leq j\leq 7\;$$

Here, $I_{ij}$ represents the integral for the i-th measurement of the j-th standard solution. $A_{ij}$ corresponds to the measured absorbance value at *t =* n. Each set of four measurements were averaged and then the results were used to plot a calibration curve. The chip was flushed with 2 mL of Milli-Q water between each standard fluid, or every four measurements. This process was repeated three times, using three different injection volumes: 50 µL, 20 µL and 4 µL. 

## 3. Results

### 3.1. Simulation and Dye Testing Results

**Figure 3:**
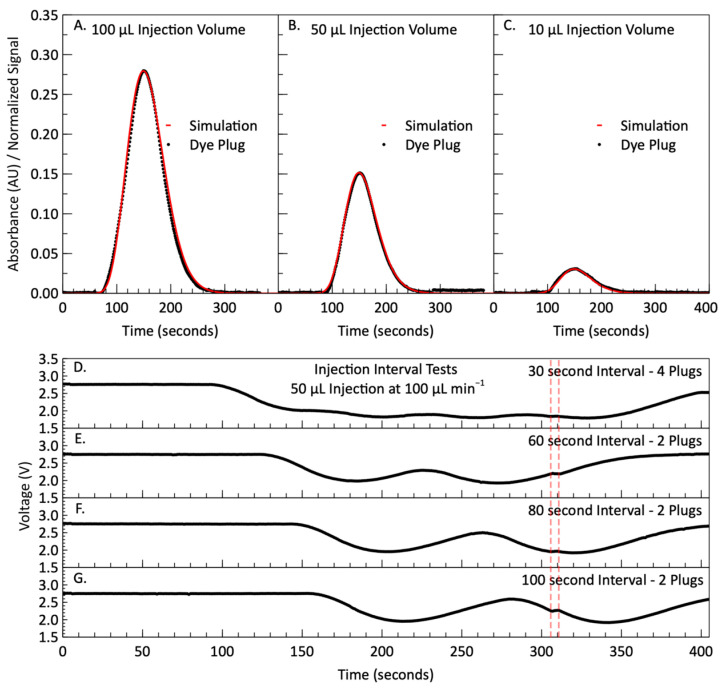
(**A**) Dispersion of a 100 µL injected dye plug after traveling 1.01 m at 100 µL min^−1^. The normalized concentration from a simulated plug that has undergone dispersion based on the Taylor–Aris simulation model is overlaid onto the absorbance signal recorded during the dispersion of a 100 µL plug of red dye. (**B**) Dispersion of a 50 µL injected dye plug after traveling 1.01 m at 100 µL min^−1^. The simulated signal is overlaid onto the recorded signal during a 50 µL injection. (**C**) Dispersion of a 10 µL injected dye plug after traveling 1.01 m at 100 µL min^−1^. The simulated signal is overlaid onto the recorded signal during a 10 µL injection. (**D**–**G**) Voltage recordings from multiple 50 µL dye injections into a 100 µL min^−1^ carrier stream. The four signals represent four different timing intervals between the end and start of each new injection: 30 s, 60 s, 80 s and 100 s. The area marked by red dashed lines shows the disturbance in the flow caused by the opening and closing of valves and the switching of directions of the syringes.

Figure 3A–C show the simulated output signal (red data points) overlaid onto the measured absorbance (black data points) for each injection volume of red dye, with a constant flow rate of 100 µL min^−1^. The simulated signals were normalized so that the peak magnitude matched the peak magnitude of the measured signal so that they could be directly compared, and it is clear that the dispersion of the dye matches very closely to the simulation. There were no reaction kinetics, and no refractive index mismatches as the dye was diluted in MilliQ, which is the same solvent as the sample carrier stream. The asymmetric nature of the curve is difficult to observe in the larger 100 µL injection volume shown in Figure 3A, but it is clear in the smaller 10 µL injection volume shown in Figure 3C.

Figure 3D–G depicts the voltages recorded during the dye injection interval testing. Figure 3D is the photodiode output from a test with four injections and only 30 s between the end of one injection and the beginning of the next. The tail ends of the dye plugs are smeared together from dispersion, and the voltage signal does not return to the original baseline value of approximately 2.8 V. In Figure 3E, we increased the timing between two injections to 60 s and the individual peaks became more distinct; however, the voltage signal does not here return to the baseline blank. Further increasing the time interval to 80 s does not improve the baseline recovery, as shown in Figure 3F. At an injection separation of 100 s (Figure 3G), the voltage returns to near blank baseline voltage. In Figure 3G, the entire curve from the 50 µL injection is visible for just under 120 s at the detector; from t = 160 to t = 280 s. The second injected plug (t = 280 to t = 400 s) demonstrates that two dye injections (50 µL or 30 s each) per syringe stroke (500 µL or 300 s) are possible. However, one dye injection per syringe stroke is used in the rest of the data to ensure the signal returns fully to baseline, corresponding to an injection interval of 150 s. This ensures that the absorbance cell is fully cleared of the previous reacted fluid and yields easily distinguishable peaks in the absorbance profiles for analysis. Furthermore, due to the solenoid valves opening and closing, the switching of the syringes causes disturbances in the steady state flow that can lead to measurement errors. This is visible in all the voltage signals in Figure 3D–G just after 300 s. By reducing the injection frequency to one per stroke, the disturbances caused by valve switching happen during the periods when there is no reaction happening within the cell, and therefore do not affect the results. 

### 3.2. Nitrite Calibration Results

**Figure 4:**
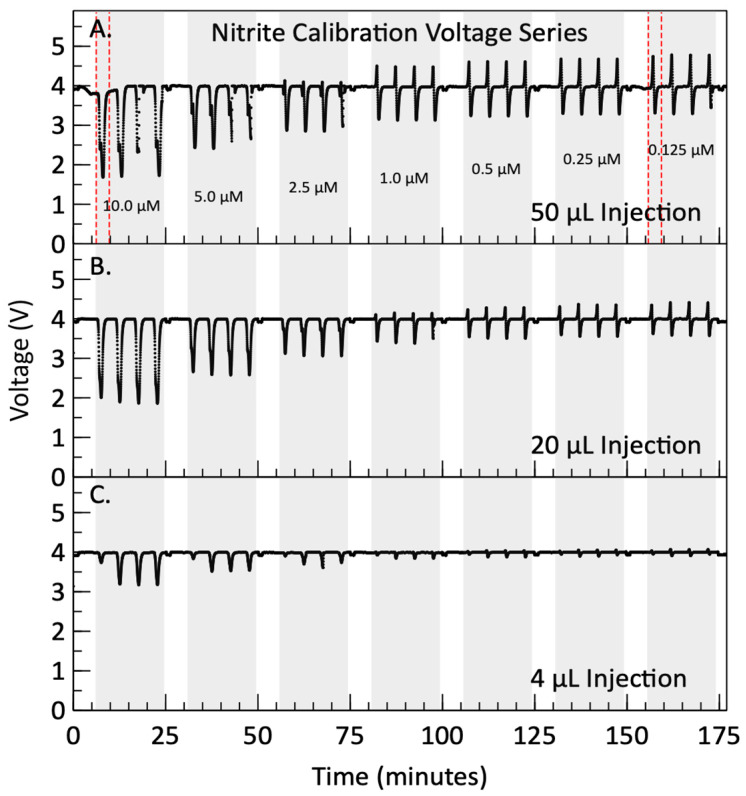
Voltage measured by the photodiode during automated nitrite calibrations with nitrite concentrations listed in µM. The areas shaded in grey represent the signal obtained during four consecutive reagent injections into a carrier stream composed of sequential standard fluids, with nitrite concentrations ranging between 0.125 µM and 10 µM. The areas shaded in white represent the rinsing between standards, where Milli-Q is flushed through the system. Panel (**A**) contains the filtered voltage from the calibration performed with 50 µL of reagent, while panels (**B**,**C**) contain the filtered voltages from the calibrations performed with 20 µL and 4 µL of reagent, respectively. The areas enclosed by dashed red lines correspond to the profiles shown in Figure 5.

After dispersion simulation and dye testing, nitrite calibration was performed by analyzing a series of seven standards ranging between 0.125 μM and 10 μM. The photodiode signal for each injection volume during the nitrite calibration is shown in Figure 4. Panels A–C display the results from the 50 µL, 20 µL and 4 µL injections, respectively. Each panel displays four repeat measurements, highlighted by the grey shaded regions, followed by a 2 mL Milli-Q wash between each standard concentration. The concentrations presented in the top panel represent the carrier stream concentrations of the prepared standards before mixing. In Figure 4C, it can be seen that the first measurement of each standard has lower absorbance compared to the other three measurements in a replicate run. This comes from the dead volume that needs to be filled in the first standard measurement after reagent syringes are primed. This behavior likely occurred in all three injection volumes, but the effect was too small to be noticeable in the larger reagent injection volume tests. Consequently, the values of the first measurements were not included in the data analysis. Beyond this “priming” artifact, two distinct phenomena are present in the data sets. 

**Figure 5:**
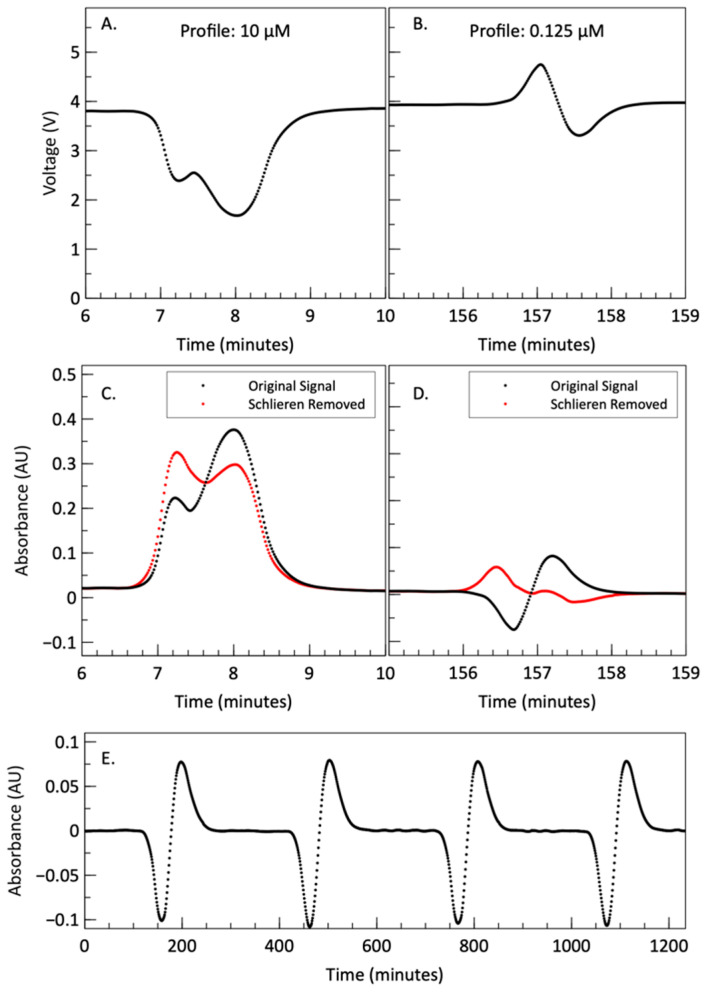
(**A**) Voltage profile for a single 50 µL reagent injection into a 10 µM carrier stream. The visible dual peaks are a result of an incomplete reaction in the center of the plug upon reaching the absorbance cell. (**B**) Voltage profile for a 50 µL reagent injection into a 0.125 µM carrier stream. The initial increase followed by a decay in voltage is a result of the Schlieren effect. The corrected absorbance profile for a single reagent injection into a 10 µM (**C**) and 0.125 µM (**D**) carrier stream. Dual peaks are still visible, but the decay and rise in signal due to the Schlieren effect are removed. (**E**) Absorbance profile of 4 consecutive 50 µL reagent injections into Milli-Q carrier stream. The rises and decays in the absorbance are the result of the Schlieren effect.

The first of the two phenomena is the “double peak” observed in the 10 µM and 5 µM standards of the 50 µL injection series. This is enhanced and displayed in Figure 5A, which is a zoomed-in profile of the first 50 µL injection into the 10 µM carrier stream shown in Figure 4A (red dashed lines). The voltage signal dips as expected, but then increases slightly before dipping again to its lowest point, and then returning to the signal of the blank. One reason could be due to an inhomogeneous degree of reaction completion along the axial plug length: less reacted at the center of the plug, and more reacted at the edges. As the reagent mixes with the carrier stream, the reaction takes place at the interfaces or ends of the injected reagent plug, progressing from the outer edges inward, causing dye formation at the tails of the reagent plug before it forms in the center. The voltage signal increases in the center of the plug because the unreacted reagent has less color, and more light is transmitted in that region. This explanation is further supported by the fact the lower volume injections, i.e., smaller plug lengths, do not display this behavior because they are able to fully react by the time they reach the detection cell. The injected plugs of 50 µL, 20 µL, and 4 µL correspondingly exhibit lengths of 200 mm (~1/5th serpentine length), 80 mm (~1/12th serpentine length), and 16 mm (~1/60th serpentine length) as they flow inside the microchannels. 

The second phenomenon is more pronounced in the lower concentrations of all three injection sets and is shown clearly in the enhanced profile shown in Figure 5B. The voltage first rises and then dips due to color development. We attribute the initial rise in voltage to the Schlieren effect, which is the lensing and focusing of light due to the interface between two fluids that have different refractive indices, shaped by the parabolic flow profile in a channel [71,72,73]. When the plug is driven through the cell using pressure-driven flow, it forms a parabolic flow profile, which forms a fluid lens due to the differences in refractive index. The reagent plug and carrier stream are composed of different fluids with slightly different refractive indices. The Griess reagent has a refractive index of 1.353 at 589 nm, while the carrier stream (nitrite stock solution) has a refractive index of approximately 1.333 at 589 nm [74], and a delta of 20 mRIU. As the reagent plug flows through the absorbance cell, light is focused towards the central axis of the detector. This results in an increase in voltage output as more light is detected by the photodiode active surface area. If the flow profile was reversed, light would be directed away from the central axis of the detector, which would result in a decrease in voltage or measured light. The reagent plug exhibits both focusing and defocusing. While the parabolic flow profile is similar on both ends of the plug, the order of refractive indices is different; i.e., the start of the plug is a reagent–carrier lens and the end of the plug is a carrier–reagent lens. Therefore, the Schlieren effect can be both additive and/or subtractive, and can produce varying degrees of refraction depending on flow conditions, namely: rate, direction, and refractive indices (density). The additive effect is notably evident for lower standard concentrations (<1 µM) with lower light absorbance (Figure 5B). However, as can be seen in Figure 5A,C, this phenomenon also affects higher concentrations by increasing the voltage (decreasing absorbance) of the front peak [71]. Given the same system and configuration used here, it is assumed that at any specific wavelength, the effect is repeatable and consistent with every injection. Therefore, it can be calibrated out as long as the absorbance signal is above the detector noise floor [73,75].

To eliminate the Schlieren effect, for each injection volume, a series of blank measurements were conducted and averaged. Since the solvent for nitrite standards is also Milli-Q water, the Schlieren effect observed when using a blank solution (0 µM nitrite) should be similar to that when solutions containing nitrite flow through the system. Therefore, by subtracting the blank signal from the sample signal, we can effectively reduce the impact of the Schlieren effect to a considerable extent. In Figure 5E, the absorbance profile of a 50 µL injection into the milli-Q carrier stream is depicted. The consecutive and repeatable absorbance troughs and peaks from successive reagent injections into a Milli-Q water carrier are attributed to the Schlieren effect. According to the Beer–Lambert law, the measured absorbances for both the sample and the blank solution can be calculated as:

(6) $$A_{b}=\log\frac{I_{ref}}{I_{b}},A_{s}=\log\frac{I_{ref}}{I_{s}}$$

where $A_{b}$, $A_{s}$, $I_{b}$ and $I_{s}$ represent the blank absorbance, sample absorbance, and intensities of the blank and sample, respectively, obtained at a wavelength of 527 nm. Additionally, $I_{ref}$ denotes the radiation intensity when the carrier stream passes through the optical cell. For each 300 s of syringe stroke, the corrected absorbance ($A_{cs}$) is calculated by subtracting the average value of four blank absorbances from the sample absorbance, as outlined below:

(7) $$A_{cs}\left[n\right]=\log\frac{\bar{I_{b}\left[n\right]}}{I_{s}\left[n\right]}=\log\left(\frac{I_{ref}\left[n\right]}{I_{s}\left[n\right]}\cdot \frac{\bar{I_{b}\left[n\right]}}{I_{ref}\left[n\right]}\right)=A_{s}\left[n\right]-\bar{A_{b}\left[n\right]},\;\;\;\;\;\;\;\;For\;1\leq n\leq 300$$

Here, $\bar{I_{b}}$ and $\bar{A_{b}}$ represent the average intensity and absorbance of the blank solution, respectively. 

In Figure 5C,D, the corrected and original absorbance profiles of a 50 µL injection into the 10 and 1.25 µM carrier streams are illustrated. Figure 5C demonstrates the effective removal of the Schlieren effect, resulting in a more symmetrical presentation of the dual peaks attributed to an incomplete reaction. Similarly, Figure 5D showcases an enhancement in absorbance measurements, with the removal of the negative peak in absorbance and the appearance of well-defined dual peaks. 

**Figure 6:**
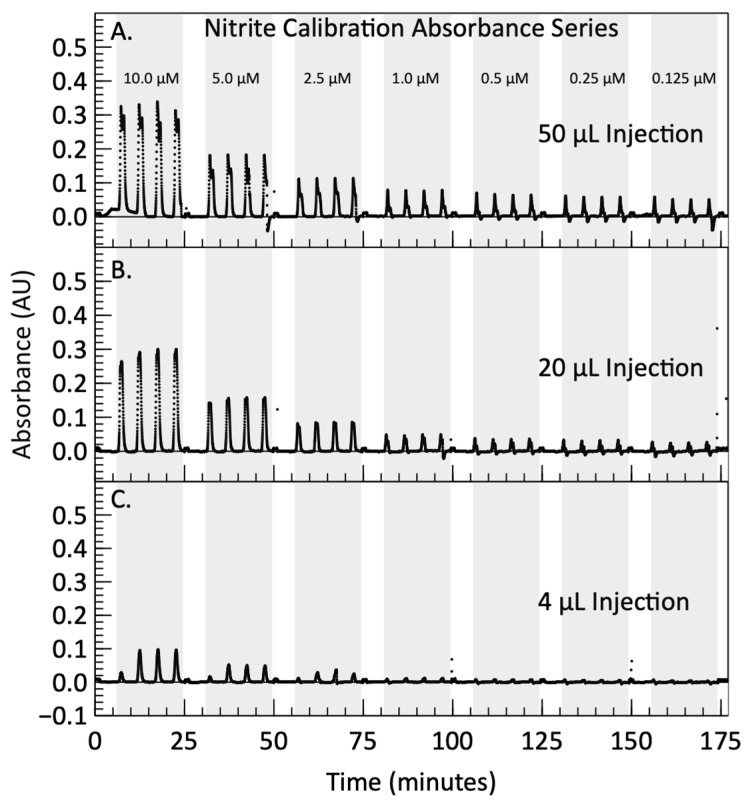
Corrected absorbance signals calculated by subtracting the averaged values of blank absorbance from the sample absorbance. A MATLAB (R2021b) script is used to integrate the absorbance signal produced by each injection, shown as the shaded colored areas between each injection peak and the time axis. Panel (**A**) contains the absorbance signal from the calibration performed with 50 µL of reagent, while panels (**B**,**C**) contain the absorbance signal from the calibrations performed with 20 µL and 4 µL of reagent, respectively.

Figure 6 shows the corrected absorbance profiles for the three nitrite calibration tests. The 50 µL injection peaks show an absorbance dip in the center, as the plugs have not yet fully mixed. The first of the 4 µL injection peaks in each standard is consistently lower than the following three. Each absorbance peak is integrated to obtain a more sensitive absorbance measurement as the reagent plug flows through the cell. Integrating absorbance over time is similar to integrating concentration over time, effectively quantifying the number of molecules that pass the detector. Since the plug is moving at a fixed flow rate, integrating over time yields a fixed volume. Consequently, the area under an absorbance curve or concentration curve serves as a direct indicator of the number of molecules present in a typical plug volume (time x fixed flow rate), and would need to be normalized by dividing out the plug volume to construct a traditional calibration curve (absorbance versus concentration).

**Figure 7:**
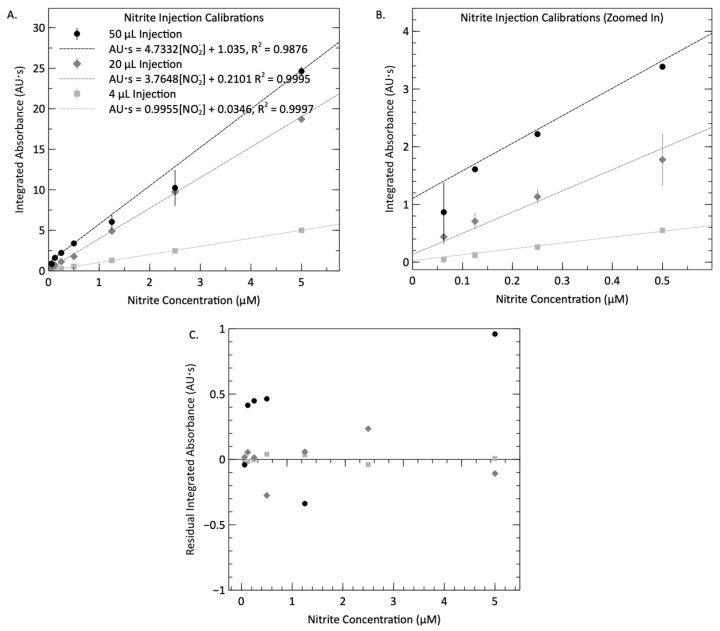
Panel (**A**) calibration curves produced using the continuous flow system, with 4 µL, 20 µL and 50 µL reagent injection volumes. The absorbance profile for each of the four consecutive reagent injections for every standard fluid is integrated and averaged to produce a resultant value, which is plotted against the final nitrate concentration. The linearity of the calibration slope for each reagent injection volume is displayed alongside the slope value and dependent variable offset. Panel (**B**) displays a closer view of the data sets with nitrite concentrations below 1 µM. Panel (**C**) shows the residual plot of the linear regressions. Measurements were performed at room temperature or 21–23 °C.

The average value for each set of triplicate integrals is plotted against the known concentration (after mixing) in Figure 7 to form a calibration curve. The mixing ratio between the injected reagent plugs and standards was 1:1, indicating that the concentrations reported in Figure 7 are half of the initially prepared standard concentrations. Figure 7B is zoomed in to show the lower concentrations of the calibration curve more clearly. For each injection volume, the relationship between the integrated absorbance and nitrite concentration was determined using linear regression. Notably, strong linearity was observed, with correlation coefficients of 0.9876, 0.9995, and 0.9997, alongside corresponding root-mean-square-error (RMSE) values of 0.86, 0.14, and 0.028 AU·s for respective injection volumes of 50, 20, and 4 µL. Although all three data sets display excellent linearity, the curve for the 50 µL injections does show more deviation from the linear fit than the other two. This is likely due to the incomplete reactions and misshapen absorbance peaks. As expected, the curve for the 50 µL injections demonstrates higher sensitivity than the other two. The slope of each regression curve reflects the sensitivity of the measurements to nitrite concentration, calculated as 4.73, 3.76, and 0.99 AU·s µM^−1^ for 50, 20, and 4 µL injections, respectively. By dividing the absorption cell length (25.4 mm) and the integration time, the corresponding attenuation coefficients are computed as 0.0116, 0.0105, and 0.0029 (µM cm)^−1^ for injection volumes of 50 µL, 20 µL, and 4 µL, respectively. The calculated attenuation coefficients for 50 µL and 20 µL injections are close to the reported values for stop–flow systems, falling within the range of 0.014–0.039 (µM cm)^−1^ [1,17,58,76,77]. Compared to our previously reported stop flow system with the attenuation coefficient of 0.0269 (µM cm)^−1^ [3], the lower coefficients, particularly noticeable in the 4 µL injection, could be attributed to the constraint imposed by dispersion in FIA systems. As indicated in [41] and shown in Figure 3A, smaller injection volumes lead to sample smearing and a decrease in absorbance, where parts of the smeared plug would be below detection limits. For the same reason, the sensitivity of 4 µL is less than those of the 50 and 20 µL injection volumes. Other factors such as variations in protocols or differences in LED emission spectra can lead to slightly different attenuation coefficients. The residual analysis of the linear regression is presented in Figure 7C. The most significant residual, measuring −1.9 AU·s, occurred at a reagent injection volume of 50 µL and nitrate standard concentrations of 5 μM. For reagent injections of 20 µL and 4 µL, the largest residuals were −0.27 AU·s (at 1 μM standard) and 0.04 AU·s (at 1 μM standard), respectively. Consequently, within the examined range of nitrate standard concentrations, accuracy errors of up to 0.4 µM (for 50 µL), 0.07 µM (for 20 µL), and 0.04 µM (for 4 µL) are observed at room temperature. One source of inaccuracy arises from the uncertainties associated with the preparation of nitrite standard solutions through a series of dilutions from a primary stock solution. For example, the propagation of errors yielded a final uncertainty of ±0.058 µM in the 10 µM standard sample. One advantage of FIA is that it permits making multiple measurements rapidly, which would enable users/operators to collect a distribution of data points about the true value, thereby improving the accuracy of the reported measurement. 

The limit of detection (LOD) was determined by using the 3-sigma method [78]. This is defined as three times the standard deviation of the blank signal. The averaged standard deviations in 15 blank measurements were calculated to be 43.2, 57.9, and 16.4 mAU·s for different injection volumes. From the slope of respective calibration curves, the LODs correspond to 54, 94, and 98 nM nitrite for 50 µL, 20 µL, and 4 µL injections, respectively. However, for a more practical assessment of the sensor’s performance, the LOQ was calculated, defined as ten times the blank standard deviation [79]. Similar to the calculations for LOD, the LOQs were determined to be 184, 312, and 328 nM nitrite for 50 µL, 20 µL, and 4 µL injections, respectively. These detection limits align with findings in the literature. For instance, Sieben et al. [1] in 2010 reported an LOD of 28 nM at sample intake (1:1 dilution, 14 nM at detector) for nitrite using a 25 mm optical path, and Luy et al. [3] in 2022 reported an LOD of 97 nM with an LOQ of 324 nM for measuring nitrite using a 25 mm inlaid optical cell. In 2007, Melchert et al. developed a flow injection technique with an LOD of 174 nM for a 10 mm path length cell. While typical nitrite levels in drinking water are in the low μM range, many aquatic environments can be below 200 nM. The system described in this work has an LOD of 54–98 nM, making it ideally suited for continuous nitrite measurement in a variety of environments. 

Considering the above system characterization, the injection of a 20 µL reagent into the sample stream yielded the best microfluidic FIA sensor performance for this chip’s configuration. This performance is evident in terms of the linearity of response within the range of 0.125–10 µM, minimal measurement error, and a comparable LOD to previous continuous and stop–flow systems [74,75]. While our system was demonstrated and characterized in the lab with simple samples (nitrite in distilled water), past research has shown that the Griess assay is robust when using artificial seawater and in situ marine measurements [80]. Future work will employ the presented system and associated optimization to enable the development of in-situ nitrite sensors for extended deployments that need to conserve reagent and attain low detection limits.

## 4. Conclusions

To advance our understanding of nutrient dynamics, there is a pressing need for automated sensors that demonstrate nanomolar detection limits with minimal reagent usage. Here, we present an automated microfluidic lab-on-chip sensor utilizing a continuous flow reagent injection method, aiming to address the challenges posed by higher reagent consumption and the reduced data sampling frequency observed in stop–flow systems. We introduced a novel variant of the inlaid absorbance cell that permits chip fabrication quality (bonding) to be verified upon visual inspection without completely obscuring the absorbance cell. The new design was demonstrated with dye tests that showed excellent agreement with the Taylor–Aris model. The design was then verified with nitrite chemistry and a calibration was conducted, whereby a series of nitrite standards ranging from 0.125 to 10 µM were evaluated with the microfluidic system. Additionally, reagent injection volumes of 4, 20, and 50 µL were systematically tested to optimize a continuous flow nitrite system. The raw voltage data revealed dual asymmetrical peaks at higher concentrations, attributed to faster chemical kinetics at the plug edge, and a rise-and-decay pattern at lower concentrations, attributed to the Schlieren effect. The Schlieren effect was effectively mitigated by subtracting the average absorbance of four blank measurements from each standard absorbance. Results from the 50 µL injection exhibited satisfactory linearity and sensitivity, although the standard deviations were somewhat elevated compared to the other two volumes. The 4 µL injection results displayed notably lower absorbances, a consequence of dispersion. The 20 µL reagent injection had the most robust performance, which compared well to the literature, and in this preliminary demonstration it was tested at 10 samples per hour. The final system was able to attain sample intake LODs of 94 nM and an LOQ of 312 nM, while only consuming 20 µL of reagent per measurement. We anticipate that after appropriate engineering, this system can be made submersible, and would provide nanomolar data whilst preserving reagent for monitoring numerous aquatic environments. 

## Figures and Tables

**Table 1:** Protocol for performing continuous flow using the system shown in Figure 1C.

Commands	Open	Close	Fill	Push	Pull
1. Open valve to draw from environment	V5	--	--	--	--
2. Set up to fill reagent syringes	V1, V2	V3, V4	--	--	--
3. Fill reagent syringes	--	--	R1, R2	--	--
4. Set up to begin flow injection	V3, V4	V1, V2	--	--	--
5. Set up push/pull configuration one	V8, V10	V9, V7	--	--	--
6. Push/pull configuration one	--	--	--	P2	P1
6.1 Delay during step 6	--	--	--	P2	P1
6.2 Reagent injection during step 6	--	--	--	R1, R2, P2	P1
7. Set up push/pull configuration two	V9, V7	V8, V10	--	--	--
8. Push/pull configuration two	--	--	--	P1	P2
8.1 Delay during step 8	--	--	--	P1	P2
8.2 Reagent injection during step 8	--	--	--	R1, R2, P1	P2
9. Repeat from step 5 until syringes are depleted					
10. Repeat from step 2 until sampling is complete					

## Data Availability

The original contributions presented in the study are included in the article, further inquiries can be directed to the corresponding authors.
